# Development of Molecularly Targeted Agents and Immunotherapies in Glioblastoma: A Personalized Approach

**DOI:** 10.1177/1179554918759079

**Published:** 2018-02-26

**Authors:** Niamh Coleman, Malaka Ameratunga, Juanita Lopez

**Affiliations:** Drug Development Unit, The Royal Marsden Hospital, London, UK

**Keywords:** Glioblastoma, high grade glioma, immunotherapy, targeted therapy

## Abstract

Over the past decade, precision cancer medicine has driven major advances in the management of advanced solid tumours with the identification and targeting of putative driver aberrations transforming the clinical outcomes across multiple cancer types. Despite pivotal advances in the characterization of genomic landscape of glioblastoma, targeted agents have shown minimal efficacy in clinical trials to date, and patient survival remains poor. Immunotherapy strategies similarly have had limited success. Multiple deficiencies still exist in our knowledge of this complex disease, and further research is urgently required to overcome these critical issues. This review traces the path undertaken by the different therapeutics assessed in glioblastoma and the impact of precision medicine in this disease. We highlight challenges for precision medicine in glioblastoma, focusing on the issues of tumour heterogeneity, pharmacokinetic-pharmacodynamic optimization and outline the modern hypothesis-testing strategies being undertaken to address these key challenges.

## Background

Glioblastoma (GBM, World Health Organization [WHO] Grade IV glioma) is the most common primary malignant tumour of the central nervous system (CNS), accounting for 12% to 15% of all intracranial tumours and 50% to 60% of gliomas.^1^ It is an aggressive and incurable disease with an annual age-adjusted incidence rate of 3.2 per 100 000 individuals in the United States^2,3^ and a median survival of only 12 to 15 months, even with optimal treatment.^4,5^ Current standard of care involves maximal safe surgical resection, followed by adjuvant chemotherapy with temozolomide combined with radiotherapy.^6,7^ Due to its infiltrative and invasive nature, the disease invariably recurs, and progression typically occurs after 6 to 9 months.^5^ On relapse, treatment options are limited, with minimal clinical efficacy,^7^ and only approximately 3% to 5% of patients survive longer than 3 years.^8^

**Figure 1.**
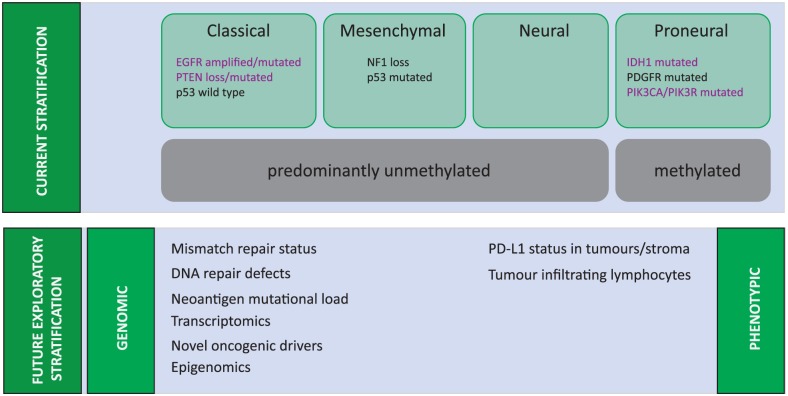
Molecular Characterisation of Glioblastoma.

Despite recent significant progress in our understanding of the molecular pathology of gliomagenesis and the epigenetics of GBM,^9^ as yet this has not translated successfully to improved clinical outcomes. There is extensive inter-patient cellular and genetic heterogeneity in GBM, but also significant intra-tumoural heterogeneity, which may contribute to therapeutic failure.^10–13^ Analysis of data from The Cancer Genome Atlas (TCGA), offering insights into genetic regulation of GBM, has led to the stratification of GBM into major molecular subgroups with recognized signaling pathways and differing prognostic significance.^14,15^ These subgroups – proneural, classical, and mesenchymal – were identified using transcriptional tumour profiling and are based on dominant genes expressed in each group (Figure 1). The classical subgroup is marked by amplifications or mutations in the epidermal growth factor receptor (EGFR) in more than 95% of cases, with high rates of concordant amplification in chromosome 7 and deletions of chromosome 10 (93%) and a complete absence of *TP53* mutations.^14,15^ The proneural subset by contrast is commonly associated with *TP53* mutations (54%) and isocitrate dehydrogenase 1 (*IDH1*) mutations, whereas the mesenchymal subtypes have a high rate of aberrations in *NF1* signalling. Overall, the TCGA data demonstrated that most GBM tumours were found to harbour alterations in common oncogenic pathways receptor tyrosine kinase (RTK) signalling through mutations/amplifications in receptors such as EGFR and PDGFRA (platelet-derived growth factor receptor A), mutations in downstream partners of AKT pathway such as PI3K and PTEN and apoptosis signalling through mutations in p53, and cell cycle control signalling through alterations in cyclin-dependent kinases.^14,15^ Indeed, 57% of GBM showed evidence of mutation, rearrangement, altered splicing, and/or focal amplification of EGFR.^14,15^

However, despite evidence of biologically distinct transcriptional profiles, the clinical relevance of these subgroups is questionable. Apart from the observation that most secondary GBMs represent the proneural subtype, the clinical outcomes of each subgroup are similar, with a slight observed survival advantage with chemo-radiotherapy in the proneural subgroup. The reality is that the impact on treatment and prognoses of these GBM subgroups is limited by genetic landscape of these tumours continually evolving at a remarkably rapid pace^16–18^ and generating an incredible degree of cellular complexity and heterogeneity within a single tumour.^19–21^ The GBM tumours are complex; they are not usually defined by a single genetic or molecular alteration. Consequently, isolating signalling pathways responsible for GBM oncogenesis has been difficult, and therapeutic outcomes from single-agent–targeted therapies have been modest.

Of course, further glioma classification systems exist, and as of the 2016 edition of the WHO classification, gliomas are classified based not only on histopathologic appearance but also on well-established molecular parameters.^22^ The incorporation of molecular features has most notably affected the classification of astrocytic and oligodendroglial tumours, which are now grouped together as diffuse gliomas, on the basis of growth pattern, behaviour, and shared *IDH1* status. Mutations in *IDH1* and, less commonly, *IDH2*, are a defining feature of most of WHO grade II and III diffuse astrocytic and oligodendroglial tumours and confer significantly improved prognosis compared with IDH wild-type tumours.^23–25^ Meanwhile, IDH wild-type GBMs, WHO grade IV, are densely cellular, pleomorphic tumours with either microvascular proliferation or necrosis, or both, and include a number of histologic variants, including giant cell GBM, gliosarcoma, and epithelioid GBM.^22^ The IDH mutant GBMs conversely comprise approximately 10% of all GBMs, and although they are histologically similar to IDH wild-type GBM, they are more likely to contain cells with oligodendroglial morphology,^22^ occur in younger adults (mean age: 45 years), and have a more favourable prognosis.^26,27^ This recent progress in the classification of the different types of glioma is indeed encouraging, and although these advances are crucial to ensure that gliomas are diagnosed and treated accurately, the hope is that these advances in classification will eventually translate into improved outcomes for patients.

The recent remarkable success of immunotherapy agents in other cancer subtypes, together with the considerable medical need in the absence of approved targeted therapies in GBM, has led to the questioning of the previously held belief that the CNS is immune privileged and thus inaccessible to anti-tumour immunity. Encouraging pre-clinical data in experimental models has led to therapies targeting immune checkpoints reaching the clinic and an invigorated interest in the immunotherapy of GBM. Here, we describe the current state of play in the development of molecularly targeted agents and immunotherapies in GBM. We summarize the data on current clinical trials for these systemic treatments in GBM and address the successes, pitfalls, and opportunities of precision medicine in this disease.

### Angiogenesis inhibition

The path to the era of personalized medicine in GBM was first paved by the recognition of O6-methylguanine-DNA methyltransferase (MGMT) hypermethylation as a valid prognostic and predictive marker in patients undergoing treatment with temozolomide.^9^ Subsequent progress in this era of molecularly targeted strategies has been characterized by promising discoveries, with a failure to translate to clinically meaningful improved outcomes for patients.

One of the initial molecularly targeted strategies for GBM was with angiogenesis inhibitors, in the light of the fact that high-grade gliomas (HGGs) are highly vascularized tumours.^28,29^ In particular, the vascular endothelial growth factor (VEGF) family of receptors have been identified as the main molecular driver of angiogenesis, although other targets including adhesion molecules, such as integrins, have also been identified.^30^ Pre-clinical studies had shown that GBMs express high levels of VEGF,^31^ with the degree of overexpression correlating with tumour aggressiveness.^32^ Several mechanisms for the potential activity of anti-angiogenic therapies in GBM have been posited including normalization of tumour vasculature^33^ and improving tumour oxygenation,^34^ thereby increasing the efficacy of chemotherapy and radiotherapy.

The initial suggestion that VEGF inhibitors may be of benefit in GBM came in 2005 when a response rate of 43% was observed in a single-arm study with bevacizumab combined with irinotecan.^35^ Subsequent studies suggested that most, if not, all of the benefits of this combination could be attributed to bevacizumab.^36^ Multiple single-arm studies subsequently confirmed unprecedented response rates in the recurrent GBM setting.^28^ These unprecedented response rates prompted accelerated Food and Drug Administration (FDA) approval for the use of bevacizumab in the recurrent setting, the commencement of 2 large clinical trials in the first-line setting, as well as the development of a host of other anti-angiogenic agents.^37–40^ Unfortunately, the initial promise of high response rates did not lead to a clear survival benefit, with a large meta-analysis demonstrating consistently improved progression-free survival (PFS) without a correlating overall survival (OS) benefit.^29^ These results have not only called into question the validity of PFS as an appropriate end point in GBM trials but have also illuminated the difficulties in neuro-imaging assessment, in particular, with the use of anti-angiogenic agents which may reduce contrast enhancement resulting in a pseudo-response.^41^ More recently, randomized data have even called into question the utility of bevacizumab in the recurrent setting, with no evidence of a survival benefit compared with chemotherapy.^42^ In addition, although bevacizumab is widely noted to have a steroid sparing effect,^28^ 2 large randomized controlled trials demonstrated discrepancies regarding the quality of life benefit of bevacizumab in the adjuvant setting.^37,38^ The lack of efficacy of bevacizumab has been mirrored in the results of other anti-angiogenic therapies in GBM, with negative trials with cilengitide, an integrin inhibitor,^43,44^ and cediranib, a small molecule pan-VEGF inhibitor.^39^

Nevertheless, despite the purported lack of survival benefit, recent efforts have focused on identifying a population of likely to derive a benefit from anti-angiogenic therapy. Sandmann et al^45^ demonstrated a survival benefit of bevacizumab in patients with proneural, IDH-1 wild-type GBM. Other markers potentially correlating with bevacizumab response include a microRNA profile,^46^ as well as imaging biomarkers such as cerebral blood volume.^47^ Although these biomarkers are promising, they are in need of clinical validation prior to more widespread adoption.

### The EGFR

More recent efforts have focused on targeting genetic alterations in GBM. The underlying genetic landscape of GBM is complex; however, there are a number of recurring alterations in the PI3K/MAPK, p53, and Rb pathways.^48^ More recently, TERT promoter alterations have also been identified as comprising a significant subset of genomic alterations in GBM.^24^ Of these pathways, alterations (mutations and/or amplifications) in EGFR are found in more than 50% of GBM^48^ and therefore represent a particularly attractive therapeutic target, particularly in the light of the clinically validated benefit of inhibition of the EGFR-mediated pathways in other tumour types.^49^ In particular, 50% to 60% of tumours found to have EGFR amplification in GBM also contain the mutant *EGFR gene*, EGFRvIII, which is a truncating mutation characterized by the deletion of exons 2 to 7.^50^ This causes an in-frame deletion of 267 amino acids in the extracellular domain, which results in functional changes leading to ligand-independent constitutive tyrosine kinase activity.^51^

Pre-clinical data supporting EGFR kinase inhibition as a viable therapeutic option, particularly in tumours co-expressing EGFRvIII and PTEN,^52^ rapidly led to the commencement of multiple clinical trials of erlotinib in GBM. Despite promising results in non-randomized studies,^53^ a large negative randomized phase II trial in the recurrent setting found a lack of discernible clinical activity.^54^ A study evaluating gefitinib, a first-generation EGFR tyrosine kinase inhibitor (TKI) after at least 5 days of continuous oral daily dosing prior to planned surgery, shed more light on the difficulties targeting this pathway in GBM.^55^ This study demonstrated that gefitinib penetrated the blood-brain barrier (BBB) and reached concentration in tumour tissue similar to that achieved in non–small-cell lung cancer (NSCLC), caused decreased phosphorylation of the EGFR, but did not significantly reduce downstream signal transducers, a finding which was replicated in a xenograft model but not in a cell line model.^55^

In part, lack of sensitivity to kinase inhibition may be due to the fact that the most common mutant found in GBM, EGFRvIII mutation, is found in the extracellular domain of the EGFR.^51,56^ Indeed, one key difference between EGFR in GBM and lung cancer is the distribution of mutations within the EGFR-coding sequence; EGFR mutations in lung cancer are located in the intracellular kinase domain, whereas EGFR mutations in GBM cluster in the extracellular domain and include in-frame deletions (such as the common EGFRvIII mutation), and missense mutations.^57^ It has been proposed instead that these GBM mutants are preferentially inhibited by EGFR inhibitors that can only be accommodated by the inactive conformation of the EGFR catalytic pocket due to their bulky aniline substituents (lapatinib).^58,59^ Given the lack of single-agent activity observed with EGFR TKIs, multiple early-phase combination trials were performed with chemotherapy; mTOR inhibitors and anti-angiogenic were also performed which failed to show any significant clinical activity.^60^

Nevertheless, given the frequent amplification of EGFR in GBM, novel therapeutic strategies targeting this pathway have recently been developed. The 2 most clinically advanced strategies have been the development of a therapeutic conjugate peptide vaccine, rindopepimut,^61^ targeting EGFRvIII, and the antibody-drug conjugate ABT-414.^47^ Rindopepimut is a peptide vaccine targeting the neo-epitope created by a 13-amino acid sequence unique to EGFRvIII, chemically conjugated to the carrier protein KLH to induce an immune response.^62^ Promising initial results^63^ culminated in the ACT III clinical trial, a single-arm study in newly diagnosed GBM, resulted in an unprecedented median OS of 21.8 months, suggesting clinical activity.^64^ These results prompted the FDA to grant breakthrough status to rindopepimut. Unfortunately, the randomized phase III study, ACT IV, failed to confirm the survival benefit of this compound; median OS with rindopepimut was 20.4 months compared with 21.1 months in the control arm^65^ (hazard ratio [HR] = 1.01; *P* = .93), with no substantial differences in PFS.

Cetuximab and nimotuzumab, both unconjugated antibodies that bind the extracellular domain of EGFR and suggested to cause internalization of EGFRvIII, have little benefits in patients regardless of their EGFR gene amplification status.^66,67^ The antibody-drug conjugate ABT-414 consists of a unique antibody targeting active EGFR or mutant EGFRvIII linked to a potent anti-microtubule agent and has shown promising results in initial phase 1 studies.^68^ Multiple phase 2 and 3 trials are currently ongoing evaluating this therapy, but it remains to be seen as to whether the elusive goal of a clinically effective therapy targeting EGFR in GBM can be achieved.

### Novel approaches

In addition to EGFR amplification, other genetic events are commonly found in GBMs. Of note, TCGA data have shown a high prevalence of mutations affecting *PTEN* in GBM.^14^ Pre-clinical data have shown a strong association between mutations in *PTEN* and reduced homologous recombination (HR) function,^69^ giving a strong pre-clinical rationale for synthetic lethality with poly-ADP ribose polymerase (PARP) inhibitors.^70,71^ This combined with possible synergy between PARP inhibition and 2 of the core components of standard GBM management, temozolomide, and radiation^72,73^ and has led to the commencement of clinical trials of PARP inhibitors in GBM which are currently recruiting.

Isocitrate dehydrogenases 1 and 2 (*IDH1* and *IDH2*) are frequently mutated in low-grade glioma (LGG) and are found in 12% of GBM; they comprise a large proportion of secondary GBM and are rarely found concomitantly with *EGFR* mutations.^27^ In glioma pathogenesis, the IDH genes are strongly correlated with the CpG island methylator phenotype, which is markedly associated with improved survival clinically.^74^ Moreover, although *IDH1* is strongly implicated in glioma pathogenesis, it has been unclear what role it plays in progression. A recent study demonstrated that IDH1/2 mutations induce an HR defect rendering tumour cells exquisitely sensitive to PARP inhibitors^75^; this IDH1-dependent PARP inhibitor sensitivity was demonstrated in a range of clinically relevant models, including primary patient-derived glioma cells in culture and genetically matched tumour xenografts in vivo, providing the basis for a possible therapeutic strategy exploiting the biological consequences of mutant IDH, rather than attempting to block 2HG production, by targeting the 2HG-dependent HR deficiency with PARP inhibition.^75^ Another recent study demonstrated in paired initial LGG tumour samples and post-progression samples that *IDH1* mutation is preserved, suggesting that it plays a role not only in tumour initiation but also in tumour maintenance.^76^ These pre-clinical data have led to the clinical development of *IDH1* inhibitors which are currently in the process of undergoing phase 1 clinical trials and have already shown promising activity.^77^

## Viral Strategies

Oncolytic viruses (OVs) are an emerging class of experimental treatments for malignant glioma, currently under investigation in the clinic, following the recent successes of talimogene laherparepvec (T-vec) in malignant melanoma.^78^ Progress, in GBM has, however, been more muted. Oncolytic viruses are live viruses that are selectively toxic to cancer cells, as well as their direct oncolytic properties; OVs are also considered a form of immunotherapy, as they can induce effective anti-viral and anti-tumour immune responses, although many of these immune-mediated mechanisms are being recognized.^79^ Several OVs have been investigated for glioma in the pre-clinical setting, including poliovirus, herpes simplex virus, adenovirus, reovirus, parvovirus, Newcastle disease virus, measles virus, and retrovirus.^80^ Although clinical trials involving OVs in GBM as single agents have largely been safe, demonstrated acceptable toxicity, and in certain studies, shown signs of efficacy by radiological evaluation and the presence of live virus in tumour biopsies a week or more after treatment,^81–83^ the overall efficacy of single-agent OV therapy has at best been modest at best.

Combination strategies involving checkpoint inhibitors are currently being explored.

CAPTIVE (NCT02798406), which explores the Combination of Adenovirus and Pembrolizumab to Trigger Immune Virus Effects, is one such study. Other OVs currently in the process of undergoing clinical trials include the oncolytic poliovirus, which uses the aberrant expression of the poliovirus receptor, CD155, in solid tumours to mediate viral cell entry.^84^

## Immunotherapy

Immunotherapy is a new paradigm in cancer care, and recent advances in the field of immune checkpoint blockade have led to dramatic results, most notably with the inhibition of the programmed cell death 1 (PD-1) and programmed cell death ligand 1 (PD-L1) interaction. Immunotherapy of HGGs has been hindered by poor definition of relevant antigens and selective measures to target the CNS, but this has evolved in recent years. Driven by the high medical need in the absence of approved targeted therapies, we now have novel neuro-oncology–specific concepts, providing new approaches, with individualized immunotherapy trials.

### CNS immunology

A major determinant of cancer pathogenesis is the interaction of tumour cells with the immune system. The CNS, in large part due to the protective nature of the BBB, was traditionally believed to be an immune-privileged site. However, the discovery that lymphatic vessels exist in the CNS^85–87^ and that immune cells can cross the BBB^88^ radically changed this assumption. Recent data indicate that leukocytes can traffic to the CNS, even in the presence of an intact BBB,^89,90^ and the flow of cerebrospinal fluid (CSF) connects the CNS to lymphatics by draining into cervical and nasal lymph nodes, providing another route for antigen and immune cell circulation.^91,92^ Taken together, these findings suggest that the immune system can combat gliomas, in addition to other tumour types.

An immune response to cancer occurs through a series of precise and stepwise actions beginning with tumour antigen presentation by antigen-presenting cells (APCs) and progressing through to priming and activation of T cells, trafficking of cytotoxic T cells (CD8+ cells) to tumours, and ultimately the killing of tumour cells.^93^ This interaction is regulated by immune checkpoints, which can be inhibitory or stimulatory. PD-1 and its ligand PD-L1 represent an inhibitory immune checkpoint at the tissue level, wherein PD-L1 expressed on tumour tissue binds PD-1 on cytotoxic T cells and leads to T-cell anergy.^94,95^ Targeting this checkpoint has proven successful in other tumour types^96–102^ and its activity in GBM is currently being explored.

In HGGs, however, it is not known whether glioma antigen cross-presentation occurs peripherally or within the CNS and is also debateable which cell types are most responsible for glioma antigen presentation. Pre-clinical models have shown that microglia are capable of cross-presenting tumour antigens to CD8-positive T cells; microglia however, even when activated express less major histocompatibility complex (MHC) and co-stimulatory markers than similarly activated dendritic cells (DCs).^103^ Tumour-infiltrating DCs, macrophages, and pericytes are also candidates for antigen presentation within the tumour bed.^104,105^ Tumour antigens could also potentially drain outside the CNS to the peripheral lymphatics for antigen presentation.

Higher grade gliomas, typically associated with BBB disruption and tumour necrosis, result in antigen expulsion and have increased numbers of immune cells throughout the tumour bed.^106^ Although higher numbers of tumour-infiltrating leukocytes may theoretically suggest a more robust immune reaction within the microenvironment of HGG vs LGGs, this does not necessarily correlate with better clinical outcomes.^107^ It is possible that, despite increased leukocyte access to HGGs, other tumour-related factors may diminish the immune response.

Generalized immunosuppression has long been an established feature in patients with GBM, and it has been well-documented that gliomas have various mechanisms to suppress the immune system. Numerous mechanisms lead to a suppressed immune response in patients with GBM.^108^ Individuals with GBM have reduced response to pro-inflammatory signals and impaired T cells with reduced proliferative potential.^108,109^ Glioma cells can also downregulate their own MHC I complexes making them invisible to immune cells,^110^ and in the presence of glioma, pro-inflammatory cytokines, such as interleukin (IL)-12, IL-18, and IFN-α, are notably reduced, whereas soluble inhibitory molecules are abundant (including IL-10, VEGF, and transforming growth factor).^103^ A subclass of DCs, plasmacytoid DCs, secrete large amounts of IFN-α in the periphery which provokes effector T-cell maturation; a recent murine study, however, demonstrated that plasmacytoid DCs within the glioma lacked IFN-α secretion and were associated with immune tolerance.^111^ Regulatory T cells (Tregs), which are thought to downregulate the immune response, have also been identified throughout gliomas, and there are data which indicate that a higher tumour-infiltrating CD8-positive T-cell/Treg ratio is clinically favourable.^112^ Furthermore, glioma cells express surface proteins that bind to leukocyte receptors – this leads to secondary signaling pathways, further dampening lymphocyte activation, such as PD-L1, which, as reported previously, leads to an increase in the Treg/effector T-cell ratio.^113^

Immunotherapeutic strategies can be broadly divided into 4 major classes: checkpoint inhibitors, adoptive strategies such as using chimeric antigen receptor (CAR) T cells, active immunotherapy such as with cancer vaccines and immune stimulatory gene therapy, and passive immunotherapies using antibodies.

### Checkpoint inhibitors

Tumours can manipulate the central function of the immune system to maintain self-tolerance and to prevent autoimmunity and thus escape immune-driven destruction. The 2 most intensely investigated co-inhibitory checkpoints in this new era of cancer immunotherapy are cytotoxic T lymphocyte–associated protein 4 (CTLA-4)/B7 and PD-1/PD-L1. CTLA-4, expressed on APCs, interacts with B7, on T cells, resulting in inhibition of clonal expansion of naïve T cells.^113^ Conversely, PD-1 on activated T cells interacts with PD-L1 expressed in target tissue to result in T-cell anergy.^112^ PD-1 has an additional ligand, PD-L2, which has limited expression. This receptor-ligand interaction, via downstream signalling, advances apoptosis of antigen-specific T cells and decreases apoptosis of Tregs.^113^ As such, the ligands for these immunosuppressive checkpoints, often overexpressed in the GBM microenvironment to inhibit T-cell response against tumour cells, have become the targets for therapies, and pre-clinical efforts aimed at inhibiting the PD-1/PD-L1 pathway have shown promising results.^113^ A pre-clinical glioma study using the GL261 mouse model, for example, demonstrated the combination of anti-PD-1 antibodies and radiotherapy doubled median OS and resulted in long-term survival in 15% to 40% of mice compared with either treatment alone.^114^

Whether this success can be replicated in the clinic is currently being addressed by a large number of ongoing clinical trials – indeed, there has been a veritable explosion in the number of clinical trials for both newly diagnosed and recurrent HGG (Table 1). Reardon et al^115^ previously presented safety and efficacy data from the CheckMate-143, a study of nivolumab alone vs nivolumab plus ipilimumab for recurrent GBM. This demonstrated that nivolumab was well tolerated with tolerability profiles consistent with observations in other tumour types, and OS was reported as an encouraging 40% at 12 months. However, 90% of patients who received combination therapy had grade 3 or 4 treatment-related adverse events (TRAEs), and 50% of patients in that arm had to discontinue treatment early due intolerability.^115^ Disappointingly, however, CheckMate-143 did not meet its primary end point of improved OS, as presented by Reardon et al^116^ at World Federation of Neuro-oncology Societies (WFNOS) 2017. The reported median OS was 9.8 months with nivolumab (95% confidence interval [CI]: 8.2-11.8) and 10.0 months with bevacizumab^116^; 12-month OS rate was 42% in both arms and PFS medians were 1.5 months with nivolumab and 3.5 months with bevacizumab.^116^ Furthermore, documented response rates were lower with nivolumab than bevacizumab, despite the more durable responses noted with nivolumab.^116^

**Table 1.** Current active checkpoint inhibitor trials listed on clinicaltrials.gov for adult patients with high-grade glioma.

Title	Registration no.	Phase	Therapy	Study design	Study population	Outcome measure
Pharmacodynamic study of pembrolizumab in patients with recurrent glioblastoma	NCT02337686	II	Pembrolizumab, surgery	Open label, single group assignment	Recurrent GBM	6-mo PFS; immune effector:Treg ratio measured at the time of surgery
Phase II study of pembrolizumab (MK-3475) with and without bevacizumab for recurrent glioblastoma	NCT02337491	II	Pembrolizumab, bevacizumab	Randomized, open label, parallel assignment	Recurrent GBM	6-mo PFS; recommended phase 2 dose/MTD
A phase I trial of hypofractionated stereotactic irradiation (HFSRT) with pembrolizumab and bevacizumab in patients with recurrent high grade gliomas	NCT02313272	I	Pembrolizumab, HFSRT, bevacizumab	Open label, single group assignment	Recurrent grade III or grade IV glioma (excluding anaplastic oligodendroglioma)	MTD
A phase I and open label, randomized, controlled phase II study testing the safety, toxicities, and efficacy of MK-3475 in combination with MRI-guided laser ablation in recurrent malignant gliomas	NCT02311582	I/II	Randomized, open label, parallel assignment	Pembrolizumab, MLA	Recurrent GBM	MTD of pembrolizumab when combined with MLA; PFS of pembrolizumab alone vs pembrolizumab plus MLA
Phase 2 study to evaluate the clinical efficacy and safety of MEDI4736 in patients with glioblastoma (GBM)	NCT02336165	II	Nonrandomized, open label, parallel assignment	Durvalumab, RT, pembrolizumab	Cohort A: newly diagnosed, unmethylated MGMT GBM; other cohorts: recurrent GBM	Clinical efficacy, as judged by survival
A proof-of-concept, pilot study of pembrolizumab (MK-3475) in patients with recurrent malignant glioma with a hypermutator phenotype	NCT02658279	Pilot	Open label, single group assignment	Pembrolizumab	Recurrent GBM, grade 3 anaplastic astrocytoma oligodendroglial tumours, grade 2 gliomas (if MRI shows contrast enhancement) with a hypermethylated phenotype	Response rate
Phase IIb trial evaluations of the effectiveness of treatment glioblastoma/gliosarcoma through the suppression of the PI3K/Akt pathway compared with MK-3475	NCT02430363	I/II	Nonrandomized, open label, parallel assignment	Pembrolizumab, suppressor of the PI3K/Akt pathways	Recurrent GBM or gliosarcoma	PFS
Phase I/II trial of radiation therapy plus temozolomide with MK-3475 in patients with newly diagnosed glioblastoma (GBM)	NCT02530502	I/II	Open label, single group assignment	Pembrolizumab, RT, TMZ	Newly diagnosed GBM	DLT of RT with TMZ and pembrolizumab, PFS
A randomized phase 2 single blind study of temozolomide plus radiation therapy combined with nivolumab or placebo in newly diagnosed adult subjects with MGMT-Methylated (tumor O6-methylguanine DNA methyltransferase) glioblastoma – CheckMate 548: checkpoint pathway and nivolumab clinical trial evaluation 548	NCT02667587	II	Randomized, double blind, parallel assignment	Nivolumab, TMZ, RT	Newly diagnosed GBM	OS
A randomized phase 3 open label study of nivolumab vs temozolomide each in combination with radiation therapy in newly diagnosed adult subjects with unmethylated MGMT (tumor O-6-methylguanine DNA methyltransferase) glioblastoma (CheckMate 498: CHECKpoint pathway and nivolumab clinical trial evaluation 498)	NCT02617589	III	Randomized, open label, parallel assignment	Nivolumab, TMZ, RT	Newly diagnosed GBM	OS
A randomized phase 3 open label study of nivolumab vs bevacizumab and multiple phase 1 safety cohorts of nivolumab or nivolumab in combination with ipilimumab across different lines of glioblastoma	NCT02017717	III	Randomized, open label, parallel assignment	Nivolumab, bevacizumab, ipilimumab	Newly diagnosed and recurrent GBM	Safety and tolerability, OS
AVeRT: anti-PD-1 monoclonal antibody (nivolumab) in combination with DC vaccines for the treatment of recurrent grade III and grade IV brain tumors	NCT02529072	I	Randomized, open label, parallel assignment	Nivolumab, pp65 DC vaccine	Recurrent WHO 3/4 glioma	Safety of administering DC vaccines with nivolumab
A pilot study to evaluate the feasibility of the combined use of stereotactic radiosurgery with nivolumab and concurrent valproate in patients with recurrent glioblastoma	NCT02648633	I	Open label, single group assignment	SRS, nivolumab, valproate	Recurrent GBM or gliosarcoma	Feasibility; incidence of adverse events

Abbreviations: DC, dendritic cell; DLT, dose-limiting toxicity; GBM, glioblastoma; HFSRT, hypofractionated stereotactic irradiation; MLA, magnetic resonance imaging–guided laser ablation; MGMT, O6-methylguanine-DNA methyltransferase; MTD, maximum tolerated dose; nivo, nivolumab; OS, overall survival; PD-1, programmed cell death 1; PFS, progression-free survival; RT, radiation therapy; SRS, stereotactic radiosurgery; TMZ, temozolomide; WT, wild type.

Reardon et al^117^ previously presented encouraging data on the single-agent activity of checkpoint inhibitor pembrolizumab at the Annual Society of Neuro-oncology (SNO) Meeting 2016. KEYNOTE-028 (NCT02054806) evaluated the safety and efficacy of the anti–PD-1 monoclonal antibody pembrolizumab in 20 advanced solid tumour types. In the GBM cohort, pembrolizumab demonstrated a manageable safety profile with grade 3-4 TRAEs observed in 15.4% of patients (lymphopenia, type 2 diabetes mellitus, arthritis, and syncope). Promising anti-tumour activity was noted; although only 1 partial response was observed, 12 patients (46%) experienced stable disease at a median duration of 39.4 weeks (95% CI: 7.1-85.9), median PFS 2.8 months (95% CI: 1.9-9.1), and median OS 14.4 months (95% CI: 10.3-not reached). Furthermore, durable response was suggested in 4 patients who continued therapy >54 weeks following enrolment.

Further encouraging preliminary safety and efficacy data from the ongoing phase 2 study of the anti–PD-L1 antibody MEDI4736 (durvalumab) (NCT02336165) were presented for the patients with recurrent bevacizumab-naïve GBM.^118^ In these 31 patients treated with durvalumab monotherapy, no grade 4/5 serious TRAEs were observed; grade 3 TRAEs were reported in 9.7%.^118^ Response rate was 13%, median PFS was 13.9 weeks (95% CI: 8.1-24.0), and 6-month PFS was 20% (90% CI: 9.7-33.0) with 5 of these 6 patients remaining progression free at 1 year.^118^ It is the durability of response in this cohort which is most exciting; all 6 patients who were progression free at 6 months remain progression free for over a year, suggesting that perhaps with this PD-L1–targeting immunotherapeutic for recurrent GBM, there is a tail of the curve which has been witnessed in other cancers – a subset of patients who are having a remarkably durable benefit. The study is also investigating immuno-correlative biomarkers with the aim of better identifying those responders.

Most of the glioma checkpoint inhibitor trials are in early phases, but 2 further phase 3 studies are assessing nivolumab in GBM: CheckMate-498 and CheckMate-548, evaluating the combination of nivolumab with radiation therapy with or without temozolomide in MGMT-unmethylated and methylated patients. Active checkpoint inhibitor trial information obtained from clinicaltrials.gov is summarized in Table 1.

The lack of survival benefit demonstrated in the CheckMate-143 trial is, of course, discouraging.^116^ A proposed hypothesis as to why gliomas display a reduced sensitivity to checkpoint inhibition alone is thought to be due to a relatively low mutational load. Checkpoint inhibition releases mutation-specific T-cell responses,^119^ and gliomas typically contain 40 to 80 non-synonymous single-nucleotide variations, which is comparatively lower than in melanoma or small-cell lung cancer, both of which tend to respond well to single-agent checkpoint inhibition.^120^ Supporting this hypothesis are the exceptional case reports of significant clinical responses to nivolumab seen in 2 siblings with biallelic mismatch repair deficiency with recurrent multifocal GBM, both of which exhibited very high mutational loads.^121^

PD-L1 is not only expressed in the tumour microenvironment of gliomas^112,122,123^ but also elevated in circulating APCs in patients with glioma.^124^ This of course may indicate biological activity, even if the therapeutic antibody does not reach sufficient intra-tumoural levels. As such, anti-PD-L1 antibodies such as atezolizumab represent an appealing strategy, where intra-tumoural or even peripheral PD-L1 expression may serve as a biomarker.^125,126^

### Chimeric antigen receptors

Chimeric antigen receptors are a novel type of adoptive T-cell transfer currently garnering interest in immuno-oncology. Chimeric antigen receptors involve the extraction of T cells from a patient and subsequently transducing the cells, using a lentiviral vector, to express a modified T-cell receptor with specific affinity to a tumour surface antigen.^127^ A weakness of adoptive T-cell transfer is that effective tumour antigen–induced T-cell activation can be hindered by weak affinity of the T-cell receptor to the peptide/MHC complex; subsequent tumour cells have a tendency to downregulate their MHC expression.^127^ The CAR-T cells are activated independent of MHC and, as such, avoid the difficulty of MHC restriction. One concern is the damage that can occur to normal tissues if the antigen expression is not tumour specific; thus, it is essential to select targets that show tumour-restricted expression.

Clinically, adoptive T-cell therapy has demonstrated its effectiveness with CAR-based treatment for B-cell malignancies,^128^ and dramatic results have been achieved in early clinical studies in relapsed acute lymphoblastic leukaemia (ALL), with one phase 1 dose escalation trial examining CD19 CAR-T cells for refractory ALL demonstrating a remarkable 70% complete response.^129^ The effects of CAR-T cells have been further investigated in renal cell carcinoma and neuroblastoma.^130–133^ In brain tumours, using CARs as a therapeutic strategy was first tested by the Jensen group, who showed that intra-tumoural delivery of IL-13 zetakine CAR eliminated orthotopic human glioma tumours in immune-compromised mice.^134^ The clinical trial assessing the safety and feasibility of this therapy in patients with recurrent GBM involved autologous cytotoxic T cells with CARs that bind to IL13Ra2 (a protein overexpressed in more than one-half of GBMs) being directly inserted into the resected tumour cavity. This therapy resulted in minimal side effects, and 2 of the 3 patients who received repeated intracranial infusions experienced transient anti-glioma immune responses.^135^ Indeed, Brown et al^136^ recently updated the results of one of these patients and reported their remarkable findings in the *New England Journal of Medicine*. In one patient who received weekly intracavitary infusions of cytotoxic T cells with CARs that bind to IL13Ra2, regression of all intracranial and spinal tumours was observed, along with corresponding increases in levels of cytokines and immune cells in the CSF.^136^ This response was sustained for 7.5 months; however, recurrence did eventually occur and preliminary results suggest that tumours downregulated IL-13α2 expression at progression.^136^

HER2-specific CAR-T cells have also been investigated, and in xenograft mouse GBM model, this led to tumour regression and a HER2-dependent anti-tumour response with increased production of IFN-γ and IL-2.^137^ A phase 1 trial is currently ongoing which will test the safety and efficacy of using HER2-specific CARs in patients with recurrent GBM (NCT02442297). The Rosenberg group at National Cancer Institute (NCI) (NCT01454596) and the University of Pennsylvania/Novartis (NCT02209376) are also testing the safety and feasibility of administering T cells expressing anti-EGFRvIII CAR to patients with gliomas expressing EGFRvIII.

The most common and severe side effect of CAR-T-cell therapy is cytokine release syndrome (CRS), a life-threatening complication involving the release of cytokines from leukocytes; this manifests clinically as fever, headache, nausea, dyspnoea, tachycardia, hypotension, and rash.^138^ The acute inflammatory reaction can cause vascular permeability and multi-organ failure; it has been observed in almost two-thirds of patients receiving CAR-T cells, typically days after the infusion. As such, although there is excitement in this developing field, the risk involved in CAR-T-cell therapy is not insignificant and, as always, recognition of adverse events is paramount, given that CRS can be rapidly reversed with corticosteroids and/or anticytokine agents.

### Cancer vaccines – active immunotherapy

With the aim of stimulating adaptive immune responses that target tumour-specific antigens, cancer vaccine strategies currently used include delivery of tumour-associated antigens, administration of tumour antigen–loaded DCs, and tumour cell vaccines.

### DC vaccination

The DC-based vaccine therapy involves the extraction of DCs from the patient, harvested in culture while being exposed to tumour lysate or particular tumour antigens, and then returned to the patient to promote a T-cell–mediated reaction. Currently, there are 2 anticipated ongoing phase 3 DC vaccine trials for newly diagnosed GBM, the most advanced using an autologous DC vaccine – DCVax-L (NCT00045968). This vaccine was investigated in 2 phase 1/2 studies^139^: 20 patients with newly diagnosed GBM and 19 with recurrent tumours received biweekly vaccines followed by monthly booster injections. The long-term survival analysis was encouraging: 33% of patients achieved a median survival of at least 48 months, and 27% achieved a median survival of at least 72 months.^139^

ICT-107 targets 6 GBM markers and is the current DC vaccine ongoing phase 3 investigation (NCT02546102). Targeting absent in melanoma 2 (AIM-2), melanoma-associated antigen 1 (MAGE-1), tyrosine-related protein 2 (TRP-2), glycoprotein 100 (gp100), HER-2, and interleukin 13 receptor a2 (IL- 13Ra2) and previous phase 2 data^140^ of ICT-107 for newly diagnosed GBM also was promising. ICT-107 was well tolerated, and it was associated with a 2-month increase in PFS and a trend towards improved OS.^140^

### Challenges

The power of molecular targeted therapy, and how to practically implement precision medicine in GBM, has been limited by diverse factors, ranging from the complex molecular biology underlying gliomagenesis to challenges such as CNS penetration of agents, target selection, and evaluation of treatment response.

First, although many agents have therapeutic potential for GBM, few of these agents have been clinically used because of concerns of its ability to penetrate the BBB and patients with brain tumours have also been historically excluded from most of the early experimental trials of novel agents. This thinking is now largely archaic, on a number of fronts. We, and others, have shown that patients with primary malignant brain tumours who meet standard strict phase 1 eligibility criteria and are enrolled onto trials of appropriately chosen compounds successfully meet phase 1 end points, such as safety and toxicity.^141^ Furthermore, surgical and radiological studies have shown that the BBB is disrupted in all patients with GBM.^142^ This has important implications clinically, as drugs that do not show pre-clinical brain penetration may in fact have utility in patients with GBM. For example, the PARP inhibitor, olaparib, penetrates both core and margins of recurrent GBM despite failing to penetrate the intact BBB^143^ and is now in phase 2 combination studies with temozolomide and radiation.^144^ In addition, as we understand the CNS cancer immunity cycle, antigen presentation and the generation of an active immune response are likely to take place peripherally within lymphatic system and as such drugs targeting various facets of the anti-cancer immune response may not need to penetrate the brain at all.

Second, as discussed in considerable detail earlier, genomic heterogeneity represents a major challenge for precision medicine in GBM. Molecular studies to date use small samples, typically one slide from initial surgical resection samples or diagnostic biopsies and are insufficient to comprehensively integrate temporal or spatial tumour evolution data. The key question arising is whether critical molecular drivers are being missed given a randomly selected single slide is used for molecular stratification at diagnosis. Treatment-mediated selective pressure is likely to subsequently facilitate the selection of the resistant clone or clones, but given the inherent risks of repeat neurosurgical procedures, patients with GBM almost never have further tissue sampling.

**Figure 2.**
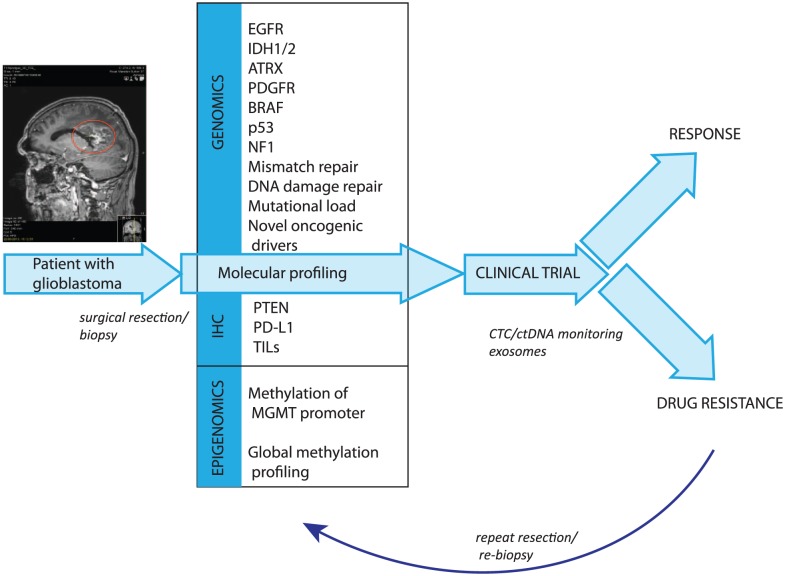
Framework for precision cancer medicine for glioblastomas.

Circulating biomarkers such as circulating-free DNA and circulating tumour cells (CTCs) are promising sources for obtaining tumour genomic material through a minimally invasive form of a liquid biopsy that can be repeated over time to account for tumour evolution and are now in use in translational clinical studies for multiple solid tumours, for example, in breast and prostate cancers.^145,146^ Circulating tumour cells from GBM tumours do cross the BBB and can be detected peripherally; work is currently ongoing to refine various platforms for their detection.^147^ Circulating tumour DNA has been reported to be more abundant than CTCs and can certainly be detected in patients with GBM where targeted next-generation sequencing (NGS) for IDH1, for example, has been performed.^148^ This poses the exciting possibility of remote monitoring of the evolution of brain tumours in response and resistance to treatment for patient care. These molecular profiles can be further complemented with the molecular analysis of nucleic acids, lipids, and proteins contained within extracellular vesicles, such as exosomes, which may contain a higher amount of clinically relevant key signalling components^149^ (Figure 2) and thus be used as a tumour biomarker for tracking cancer progression and as a potential therapeutic target/delivery system. Given that, intriguingly, exosomes may play a role in a range of biological processes within the progression of GBM,^150,151^ it is no surprise that targeting exosome-mediated cellular interactions is becoming an area of interest for therapeutics. Indeed, DC-derived exosomes appear to express both MHC class I and II, and given the role of exosomes in modulating immune response, the appliance of immunotherapy using exosomes for the treatment of gliomas, while still in its infancy,^152^ is a thought-provoking concept.

Prioritizing the numerous available therapies, and biomarkers that may be detected, requires creative efficient clinical testing platforms. INSIGhT (INdividualized Screening Trial of Innovative GBM Therapy) (NCT02977780) is the first GBM umbrella trial where patients are assessed for multiple pre-specified genetic aberrations using NGS or other platforms and then either randomized to standard therapies or matched to biomarker-based targeted treatment arms agents that are currently ongoing.^153^

The greater challenge moving forward is how to integrate the potentially complementary fields of both targeted therapies and immunotherapies, to improve precision cancer treatments for patients with GBM. Emerging biology is unravelling the myriad of ways in which tumour oncogenic drivers can modulate the tumour microenvironment, and how targeted therapies can therefore affect the host immune response.^147^ For example, PTEN loss has been shown to increase PD-L1 expression in gliomas^148^ and has also been associated with resistance to immune checkpoint inhibitors in other tumours’ settings,^154^ supporting the evaluation of combinatorial strategies targeting the PI3K-AKT pathway to increase the efficacy of immunotherapy. The interaction between EGFR-driven cancers and the immune system is much less clear, with patients with NSCLC harbouring EGFR mutations having poor outcomes with immunotherapy (Table 2).^155^

**Table 2.** Outcomes of clinical trials in molecularly targeted agents and immunotherapies in glioblastoma.

Title	Authors	Phase	Therapy	Study population	Outcome	Positive/negative study	Reference
*Angiogenesis inhibition*							
Phase II trial of single-agent bevacizumab followed by bevacizumab plus irinotecan at tumour progression in recurrent glioblastoma	Kreisl et al	II	Bevacizumab	Recurrent GBM	The 6-mo PFS was 29% (95% CI: 18%-48%). The 6-mo OS was 57% (95% CI: 44%-75%). Median OS was 31 wk (95% CI: 21-54 wk). Early MRI response was predictive of long-term PFS	Positive	36
AVAglio: Phase 3 trial of bevacizumab plus temozolomide and radiotherapy in newly diagnosed glioblastoma multiforme	Chinot et al	III	Bevacizumab	Newly diagnosed GBM	OS did not differ significantly between groups. Longer PFS in the bev group (10.6 vs 6.2 mo; HR for progression or death, 0.64; 95% CI: 0.55-0.74; *P* < .001)	Negative	37
A randomized trial of bevacizumab for newly diagnosed glioblastoma	Gilbert et al	III	Bevacizumab	Newly diagnosed GBM	No significant difference in the duration of OS between the bevacizumab group and the placebo group (median, 15.7 and 16.1 mo; HR in bev group, 1.13). PFS was longer in the bev group (10.7 vs. 7.3 mo; HR for progression or death, 0.79)	Negative	38
Phase II study of cediranib, an oral pan-vascular endothelial growth factor receptor tyrosine kinase inhibitor, in patients with recurrent glioblastoma	Batchelor et al	II	Cediranib	Recurrent GBM	PFS-6 was 25.8%. Radiographic PR was observed by MRI in 17 (56.7%) of 30 evaluable patients	Positive	39
Cilengitide combined with standard treatment for patients with newly diagnosed glioblastoma with methylated MGMT promoter (CENTRIC EORTC 26071-22072 study): a multicentre, randomised, open-label, phase 3 trial	Stupp et al	III	Cilengitide	Newly diagnosed GBM	None of the predefined clinical subgroups showed a benefit from cilengitide	Negative	44
Randomized phase II study of cilengitide, an integrin-targeting arginine-glycine-aspartic acid peptide, in recurrent glioblastoma multiforme	Reardon et al	II	Cilengitide	Recurrent GBM	Anti-tumour activity was observed in both treatment cohorts; 6-mo PFS of 15% and a median OS of 9.9 mo	Positive	40
Phase III trial exploring the combination of bevacizumab and lomustine in patients with first recurrence of a glioblastoma: the EORTC 26101 trial	Wick et al	III	Bevacizumab + lomustine	Recurrent GBM	OS was not superior in the bev arm (HR: 0.95; CI: 0.74-1.21), *P* = .650; PFS was longer with the addition of bev to lomustine (HR: 0.49; CI: 0.39-0.61)	Negative	42
Patients with proneural glioblastoma may derive overall survival benefit from the addition of bevacizumab to first-line radiotherapy and temozolomide: Retrospective analysis of the AVAglio trial	Sandmann et al	III	Bevacizumab	Newly diagnosed GBM	A multivariable analysis revealed that bev conferred a significant OS advantage vs placebo for patients with proneural IDH1 WT tumours (17.1 vs 12.8 mo, respectively; HR: 0.43; 95% CI: 0.26-0.73; *P* = .002)	Positive	45
*EGFRvIII-directed*							
A phase II, multicenter trial of rindopepimut (CDX-110) in newly diagnosed glioblastoma: the ACT III study	Schuster et al	II	Rindopepimut (CDX-110)	Recurrent GBM	PFS at 5.5 mo (∼8.5 mo from diagnosis) was 66%. mOS was 21.8 mo, and 36 OS was 26%	Positive	64
ACT IV: An international, double-blind, phase 3 trial of rindopepimut in newly diagnosed, EGFRvIII-expressing glioblastoma	Weller et al	II	Rindopepimut (CDX-110)	Recurrent GBM	Median OS with rindopepimut was 20.4 mo compared with 21.1 mo in the control arm	Negative	65
*IDH1 inhibitor*							
Ag120, a first-in-class mutant IDH1 inhibitor in patients with recurrent or progressive Idh1 mutant glioma: results from the phase 1 glioma expansion cohorts	Mellinghoff et al	I	Ag120	Recurrent GBM	AEs (>10%) regardless of attribution were mostly grade 1/2; headache (4.5% grade 3), nausea, vomiting, fatigue, and diarrhoea	Positive	77
*Viral strategies*							
A phase 1 trial of oncolytic HSV-1, G207, given in combination with radiation for recurrent GBM demonstrates safety and radiographic responses	Markert et al	I	G207	Recurrent GBM	Treatment was well tolerated. 3 instances of marked radiographic response to treatment occurred. The median survival time from G207 inoculation until death was 7.5 mo (95% CI: 3.0-12.7)	Positive	83
*Checkpoint inhibitors*							
Safety and activity of nivolumab (nivo) monotherapy and nivo in combination with ipilimumab (ipi) in recurrent glioblastoma (GBM): Updated results from CheckMate-143	Reardon et al	I	Nivo monotherapy and nivo + ipi	Recurrent GBM	Tolerability profiles in pts receiving nivo and nivo + ipi were consistent with observations in other tumour types, with no new safety signals	Positive	115
OS10.3. Randomized phase 3 study evaluating the efficacy and safety of nivolumab vs bevacizumab in patients with recurrent glioblastoma: CheckMate 143	Reardon et al	III	Nivo vs Bev	Recurrent GBM	Nivo did not demonstrate an improved OS compared with bev. The ORR was lower with nivo than bev; however, responses with nivo were more durable	Negative	116
ATIM-35. Results of the phase IB keynote-028 multi-cohort trial of pembrolizumab monotherapy in patients with recurrent PD-L1-positive glioblastoma multiforme (GBM)	Reardon et al	IB	Pembro monotherapy	Recurrent GBM	Manageable safety profile, and consistent with that of other PD-1 agents, promising anti-tumour activity in patients with recurrent GBM	Positive	117
ATIM-04. Phase 2 study to evaluate the clinical efficacy and safety of medi4736 (durvalumab [dur]) in patients with glioblastoma (GBM): results for cohort B (dur monotherapy), bevacizumab (bev) Naïve patients with recurrent GBM	Reardon et al	I	Durvalumab	Recurrent GBM	Manageable toxicities. Response rate was 13%, median PFS was 13.9 wk (95% CI: 8.1-24.0), and 6-mo PFS was 20% (90% CI: 9.7-33.0) with 5 of these 6 patients remaining progression free at 1 y	Positive	118
*DC vaccination*							
A randomized, double-blind, placebo-controlled phase 2 trial of dendritic cell (DC) vaccination with ICT-107 in newly diagnosed glioblastoma (GBM) patients	Wen et al	II	DC vaccination with ICT-07	Newly diagnosed GBM	ICT-107 was safe and well tolerated and it was associated with a 2-mo increase in PFS (*P* = .02 two-sided, HR = 0.56) and a trend towards improved OS	Positive	140
*PARP inhibitor*							
Results of the OPARATIC trial: A phase I dose escalation study of olaparib in combination with TMZ in patients with relapsed GBM	Halford et al	I	Olaparib	Recurrent GBM	Olaparib penetrates both core and margins of recurrent GBM despite failing to penetrate the intact brain barrier in pre-clinical healthy rodent models. Combination with extended low dose TMZ is safe and well tolerated, yielding encouraging 6 mo PFS rates	Positive	143

Abbreviations: AEs, adverse events; CI, confidence interval; GBM, glioblastoma; HR, hazard ratio; ipi, ipilimumab; nivo, nivolumab; ORR, objective response rate; OS, overall survival; PD-1, programmed cell death 1; pembro, pembrolizumab; PFS, progression-free survival; PR, partial response; TMZ, temozolomide; WT, wild type; mOS, meaning median overall survival.

## Conclusions

In this era of precision medicine, the sluggish progress in the advancement of therapy in GBM is insupportable. Results from single-agent–targeted therapy trials have been modest, and the success of single-agent immunotherapeutic agents to date has been mixed, although encouragingly there are a multitude of ongoing trials.

Future successes in molecularly targeted agents and immunotherapies in neuro-oncology will likely depend on the development of rationally designed combination trials – trials incorporating both surgical arms, allowing for further tumour molecular characterization and creative biomarker selection and development. However, given the innumerable permutations of possible combination regimens with targeted agents, chemotherapy, radiation, and immunotherapy, a deep understanding of the cancer biology of GBM and its interaction with the immune system must underpin robust biology-driven approaches.

Glioblastoma tumours are profoundly complex. Although there is unlikely to be a single ‘magic bullet’ for GBM, there is much to be hopeful about as we focus on innovative biomarker-driven trial designs with greater collaborations between academic and industry partners to truly achieve precision medicine for GBM.
